# Combined Metabolome and Transcriptome Analyses Reveal the Effects of Mycorrhizal Fungus *Ceratobasidium* sp. AR2 on the Flavonoid Accumulation in *Anoectochilus roxburghii* during Different Growth Stages

**DOI:** 10.3390/ijms21020564

**Published:** 2020-01-15

**Authors:** Ying Zhang, Yuanyuan Li, Xiaomei Chen, Zhixia Meng, Shunxing Guo

**Affiliations:** Institute of Medicinal Plant Development, Chinese Academy of Medical Sciences & Peking Union Medical College, Beijing 100193, China; zhangying908@163.com (Y.Z.); liyuanyuan184@163.com (Y.L.); cxm_implad@163.com (X.C.)

**Keywords:** *Anoectochilus roxburghii*, *Ceratobasidium* sp., metabolome and transcriptome analyses, flavonoid, HPLC-MS/MS, qRT-PCR

## Abstract

*Anoectochilus roxburghii* is a traditional Chinese herb with high medicinal value, with main bioactive constituents which are flavonoids. It commonly associates with mycorrhizal fungi for its growth and development. Moreover, mycorrhizal fungi can induce changes in the internal metabolism of host plants. However, its role in the flavonoid accumulation in *A. roxburghii* at different growth stages is not well studied. In this study, combined metabolome and transcriptome analyses were performed to investigate the metabolic and transcriptional profiling in mycorrhizal *A. roxburghii* (M) and non-mycorrhizal *A. roxburghii* (NM) growth for six months. An association analysis revealed that flavonoid biosynthetic pathway presented significant differences between the M and NM. Additionally, the structural genes related to flavonoid synthesis and different flavonoid metabolites in both groups over a period of six months were validated using quantitative real-time polymerase chain reaction (qRT-PCR) and high-performance liquid chromatography coupled with tandem mass spectrometry (HPLC-MS/MS). The results showed that *Ceratobasidium* sp. AR2 could increase the accumulation of five flavonol-glycosides (i.e., narcissin, rutin, isorhamnetin-3-*O*-beta-d-glucoside, quercetin-7-*O*-glucoside, and kaempferol-3-*O*-glucoside), two flavonols (i.e., quercetin and isorhamnetin), and two flavones (i.e., nobiletin and tangeretin) to some degrees. The qRT-PCR showed that the flavonoid biosynthetic genes (*PAL*, *4CL*, *CHS*, *GT*, and *RT*) were significantly differentially expressed between the M and NM. Overall, our findings indicate that AR2 induces flavonoid metabolism in *A. roxburghii* during different growth stages, especially in the third month. This shows great potential of *Ceratobasidium* sp. AR2 for the quality improvement of *A. roxburghii*.

## 1. Introduction

*Anoectochilus roxburghii* (Wall.) Lindl., also called “Jin Xianlian” and “Jin Xianlan” is a perennial herb of the genus *Anoectochilus* of the Orchidaceae family. It is widespread in southern China and considered a famous drug in the provinces of Jiangxi, Taiwan, Guangdong, Guizhou, Zhejiang, and Fujian which is its main markets [1]. The herb is a valuable Chinese medicinal material that is known as the “King Medicine”, the “Golden Grass”, and the “Bird Ginseng” by countryfolk [2]. The whole plant is used as a medicine and has the efficacy to clear the heat, cool the blood, eliminate dampness, and detoxification. Many pharmacological studies have demonstrated its wide range of pharmacological effects including its antioxidant [3,4], hepatoprotective [3], anti-diabetic [5,6], anti-rheumatoid arthritis [7], anti-hyperglycemic [4,8], anti-inflammatory [9] and many other properties. Due to the fact of its high medical values, *A. roxburghii* is not only widely used in medicine and health care but also in beauty and drinking products with an increasing market demand. The average annual demand in South Korea and Japan is more than 1000 t, 70% of which depend on imports [10]. The vigorous market demand results in excessive harvesting and exploitation of the plant, leading to resource depletion. Thus, tissue culture has become the main source of commercial *A. roxburghii*.

Most orchids form mycorrhizae with mycorrhizal fungi [11]. The fungal hyphae form coiled structures termed “peloton” inside the cells of the plant roots which play a key role in the nutrients’ exchange and absorption between the orchid and its mycorrhizal fungi [12]. The process is unique and complex involving various processes related to growth and development, such as colonization, increasing the survival rate and morphological growth. Moreover, the mycorrhizal fungal elicitor can rapidly induce the expression of relative genes that are related to secondary metabolic pathways which result in a significant accumulation of active ingredients in the host plant [13]; this was demonstrated in orchid by a few studies. The symbiosis between *Dendrobium nobile* and *Mycena* sp. MF23 caused the accumulation of dendrobine and polysaccharide [14,15]. *Mycena* sp. MF23 could also stimulate the accumulation of flavonoid and kinsenoside in *A. formosanus* [16]. For *A. roxburghii*, the major bioactive components include polysaccharides, kinsenosides, steroids, triterpenes, amino acids, alkaloids, and flavonoids that have been regarded as the quality standard of *A. roxburghii* [10]. However, *A. roxburghii* contains a very limited number of flavonoids, which limits the development and utilization of *A. roxburghii*-based medicines. In order to improve the content of flavonoid in *A. roxburghii* and to avoid excessive exploitation, many methods have recently been put forward by researchers [17,18,19]; the role of mycorrhizal fungi in the accumulation of flavonoid in *A. roxburghii* has been gradually recognized. Wang et al. reported that flavonoid accumulated significantly in *A. roxburghii* growth for 8 weeks treated with different fungi such as *Rhizoctonia* sp. cw-6 and cw-13, *Exophila pisciphila* (cw-8), *Nemania* sp. (cw-10), and *Umbelopsis* sp. (cw-1) [20]. However, few studies investigated the effects of mycorrhizal fungi on flavonoid accumulation in *A. roxburghii* during different growth stages.

The flavonoid biosynthetic pathway has been well characterized in some medicinal plants such as *Gnetum parvifolium* [21], *Chrysanthemum morifolium* [22], *Lotus japonicus* [23]. Its biosynthesis can be divided into two stages: phenylpropanoid and flavonoid pathways. Phenylalanine ammonia-lyase (PAL) is the first enzyme of the phenylpropanoid pathway which can convert phenylalanine into cinnamic acid [24]. Cinnamic acid is then converted into p-coumaric acid by trans-cinnamate 4-hydroxylase (C4H). Next, 4-coumarate CoA ligase (4CL) converts coumaric acid into its CoA ester. 4CL is one of the key branch point enzymes in the phenylpropanoid pathway and its products are subsequently used by various oxygenases, reductases, and transferases for the biosynthesis of lignin, flavonoids, anthocyanins, aurones, stilbenes, coumarins, suberin, cutin, and sporopollenin [25]. Chalcone synthase (CHS) and chalcone isomerase (CHI) are involved in two step condensation reaction, producing naringenin chalcone and naringenin, respectively. Then, flavanone is catalyzed by flavonoid 3′-hydroxylase (F3′H) and other enzymes. Subsequently, flavanone produces the branches of flavone and dihydroflavonol under the catalysis of flavone synthase (FNS) and flavanone 3-hydroxylase (F3H), respectively. Next, flavonol synthase (FLS) catalyzes C-3 hydroxylation in the structure of dihydroflavonols to form various flavonols, and flavonol-glycosides are formed by flavonoid 3-*O*-glucosyltransferase (GT) and rhamnosyltransferase (RT) or GT.

With the rapid development of high-throughput sequencing technology and systems biology, multi-omics technology has become an indispensable research method in the field of life science [26,27]. It can provide the dynamic changes of the plant’s growth and development from the cell to the individual level. The metabolome is a powerful approach to qualitatively and quantitatively analysis all the small-molecule metabolites (mass ≤ 1000 Da) in the cells or tissues of an organism at any physiological period using different analysis technologies including nuclear magnetic resonance (NMR) spectroscopy, liquid chromatograph-mass spectrometer (LC-MS), and gas chromatography-mass spectrometer (GC-MS) [28]. In other words, this method could provide the global metabolic changes. Similarly, the transcriptome means the detection of all RNA transcripts in a sample and reflects gene expression differences between different treatments [29]. Integrated transcriptome and metabolome analyses have been successfully applied to study the metabolic pathways of some substances [30,31], the color formation of vegetables, fruits, and flowers [32,33], the stress resistance mechanisms [34,35], and the growth and development mechanisms of the plants [36,37]. The combination can not only elucidate changes in the content of a series of metabolites, but it can also analyze the corresponding differentially expressed genes.

To investigate the changes of metabolites in *A. roxburghii* that is infected with mycorrhizal fungus, we performed metabolome and transcriptome analyses on six-month growth data of the mycorrhizal *A. roxburghii* (M) and non-mycorrhizal ones (NM). The results indicated that AR2 significantly promoted flavonoid biosynthesis in the plant. During the growth stage, the flavonoid content (two flavones: nobiletin and tangeretin; two flavonols: quercetin and isorhamnetin; five flavonol-glycosides: narcissin, rutin, isorhamnetin-3-*O*-beta-d-glucoside, quercetin 7-o-glucoside, and kaempferol 3-*O*-glucoside) in different metabolites and the expression of the genes that were related to the biosynthesis of flavonoid were further tested over a period of six months using high-performance liquid chromatography coupled with tandem mass spectrometry (HPLC-MS/MS) then quantitative real-time polymerase chain reaction (qRT-PCR). These will provide valuable information to reveal the effects of *Ceratobasidium* sp. AR2 on the flavonoid accumulation in *A. roxburghii*.

## 2. Results

### 2.1. Detecting Mycorrhizal Fungus Colonization in A. roxburghii

**Figure 1:**
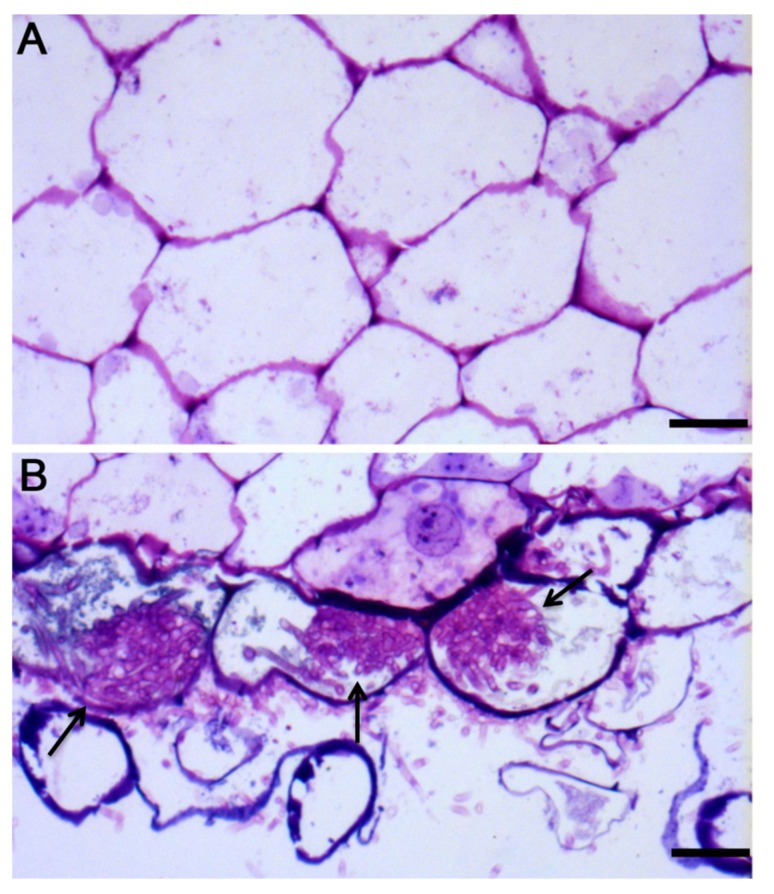
The semi-thin sections of root of *A. roxburghii* after two weeks of symbiotic cultivation. (**A**) the control, scale bar = 50 mm; (**B**) the treatment; arrows represent pelotons, scale bar = 50 mm.

Pelotons (hyphae coils) formation is the important characteristic of orchid mycorrhizal association [38]. In the study, *Ceratobasidium* sp. AR2 would soon grow over the whole substrate for about two weeks. At this time, both morphological observation and toluidine blue staining, which showed no hyphae in the control group (Figure 1A) and that the intracellular pelotons existed in the treatment (Figure 1B), indicated that a symbiotic relationship had been established between *A. roxburghii* and mycorrhizal fungus *Ceratobasidium* sp. AR2.

### 2.2. Identification of Metabolites

**Figure 2:**
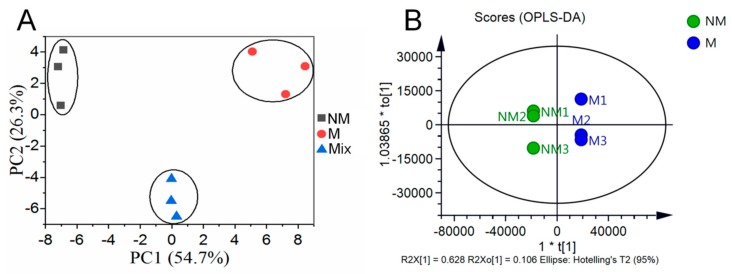
PCA and OPLS-DA scores plots derived from ultra-performance liquid chromatography-electrospray ionization-tandem mass spectrometry (UPLC-ESI-MS/MS) profiling of non-mycorrhizal *A. roxburghii* (NM) and mycorrhizal *A. roxburghii* (M) growth for six months. (**A**) PCA scores plot of the two samples (NM and M growth for six months) and the quality control sample (mix, the same volume of sample extract from the NM and M growth for six months was prepared by mixing); the x-axis represents the PC1 and the y-axis represents PC2. (**B**) OPLS-DA scores plot of the putatively annotated metabolites from NM and M growth for six months. The x-axis represents the score value of main components in the orthogonal signal correction process and the differences between the groups can be seen from the direction of the x-axis; the y-axis represents the scores of orthogonal components in the orthogonal signal correction process and the differences within the groups can be seen from the direction of the y-axis.

The M and NM growth for six months were used for metabolome analysis. Overlapping analysis of the total ion chromatography (TIC) in different quality control (QC) samples showed that the retention time and peak intensities were consistent (Figure S1), which indicated that the instrument had a good stability and could thus be used for subsequent analysis. Principal component analysis (PCA) was performed on the NM, M, and mixed samples. Principal component 1 (PC1) and principal component 2 (PC2) were 54.7% and 26.3%, respectively (Figure 2A). The metabolite profiles of *A. roxburghii* were then subjected to orthogonal partial least squares discriminant analysis (OPLS-DA). The result showed that the R2X, R2Y, and Q2 were 0.862, 1.000, and 0.995, respectively (Figure 2B), which indicated the model of OPLS-DA was stable and reliable. The score plots of PCA and OPLS-DA exhibited an obvious separation between the M and NM, and each formed a cluster. These suggested that mycorrhizal fungus AR2 affected the metabolism in *A. roxburghii*.

**Figure 3:**
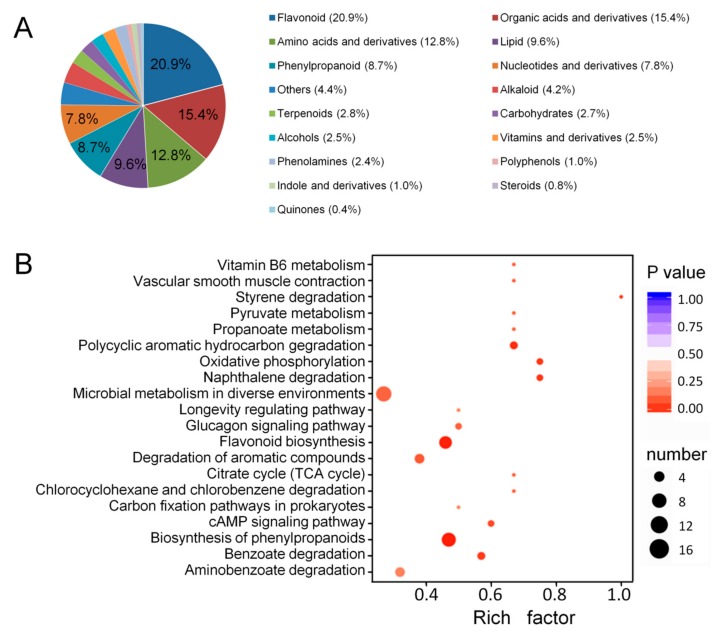
Component analysis of the putatively annotated metabolites and pathway enrichment analysis of the DAMs. (**A**) Component analysis of the putatively annotated metabolites from non- mycorrhizal *A. roxburghii* (NM) and mycorrhizal *A. roxburghii* (M) growth for six months. The percentage of the top six metabolites are shown in the graph. The percentage after each compound represents the percentage of the number of DAMs of a certain class of compounds in the total DAMs. (**B**) Pathway (top 20) enrichment analysis of the DAMs between the NM and M growth for six months. The x-axis represents the corresponding rich factor of each pathway. The y-axis represents the name of pathway. The color of the dot is *p*-value, and the closer it is to 0, the more significant the enrichment is. The size of the point represents the number of DAMs enriched in the corresponding pathway. The rich factor is the ratio of the number of metabolites in the corresponding pathway to the total number of metabolites detected and annotated in the pathway. The higher the value of rich factor is, the higher the enrichment degree is.

A total of 709 metabolites with known structures were identified in M and NM under quality validation, each of which was analyzed using three biological replicates. Detailed information about the identified metabolites, including the compounds, classes, molecular weights, ionization models, Kyoto encyclopedia of genes and genomes (KEGG) pathways, and quantities for each of the three periods is shown in Table S1. Flavonoid (20.9%), organic acids, and derivatives (15.4%), amino acids and derivatives (12.8%), lipid (9.6%), and phenylpropanoid (8.7%) accounted for a large proportion of these 709 metabolites (Figure 3A).

### 2.3. Identification of Differentially Accumulated Metabolites and Differentially Accumulated Flavonoids

Differentially accumulated metabolites (DAMs) were defined as those exhibiting a fold change ≥ 2 or ≤0.5 and a variable importance of projection (VIP) ≥ 1. In total, 135 DAMs were identified, among which 63 and 72 metabolites were upregulated and downregulated, respectively. To further understand the DAMs function and the related biological processes they participated in, pathway enrichment analysis of the DAMs was conducted using KEGG. The results showed that the terms “Biosynthesis of phenylpropanoids”, “Flavonoid biosynthesis”, “Polycyclic aromatic hydrocarbon degradation”, “Oxidative phosphorylation”, and “Naphthalene degradation” were significantly enriched (Figure 3B). Among them, the flavonoid biosynthesis began with the biosynthesis of phenylpropanoids. To some extent, these data further indicated that the flavonoid metabolism in *A. roxburghii* was significantly affected by mycorrhizal fungus AR2.

Furthermore, 148 metabolites involved in the flavonoid metabolism were identified, among which nine metabolites that belonged to two flavones (i.e., nobiletin and tangeretin), two flavonols (i.e., quercetin and isorhamnetin) and five flavonol-glycosides (i.e., narcissin, rutin, isorhamnetin-3-*O*-beta-d-glucoside, quercetin-7-*O*-glucoside, and kaempferol-3-*O*-glucoside) were obviously different between M and NM.

### 2.4. Transcriptome Profiles of Mycorrhizal and Non-Mycorrhizal A. roxburghii

The expression profiles of M and NM grown for six months were also analyzed using RNA-sequencing (RNA-seq). The Q20 and Q30 base percentages were greater than or equal to 97.79% and 93.61%, respectively (Table 1). The GC contents of the M and NM were in the range of 48.10–49.06% and 47.93–48.43%, respectively (Table 1). Compared with the control group, 4341 genes were shown to be differentially expressed, including 2915 upregulated genes and 1426 downregulated genes.

**Figure 4:**
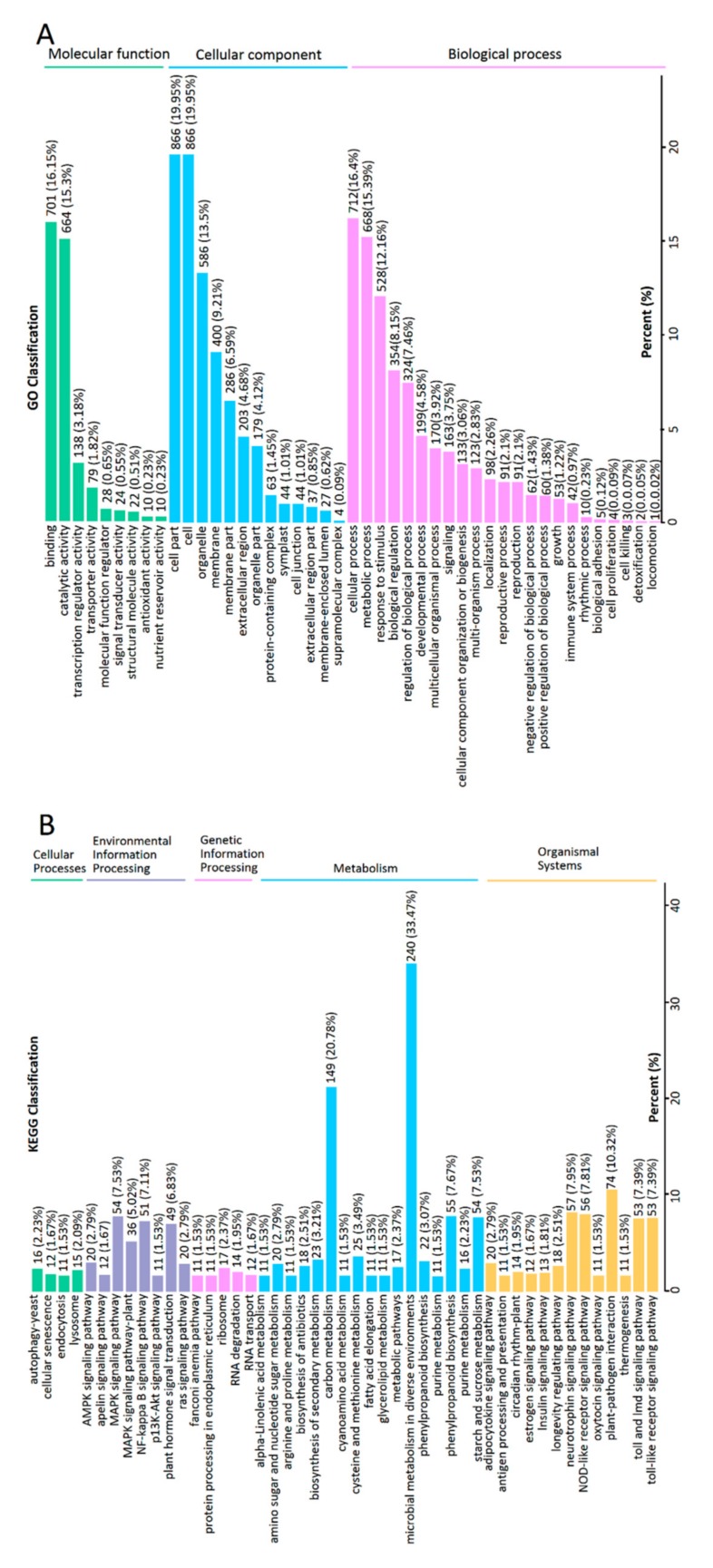
The classification column of GO and KEGG from the DEGs between the NM and M growth for six months. (**A**) GO classification of DEGs. The x-axis represents the secondary GO item. The y-axis represents the proportion of the DEGs in the total number of DEGs. The labels above the columns is the number and proportion of DEGs of this GO item. (**B**) KEGG classification of DEGs. The x-axis represents the name of KEGG pathway. The y-axis represents the proportion of genes annotated to the pathway in the total of annotated genes. The labels above the columns represent the classification of KEGG pathway.

Genes with |log_2_(fold change)| > 1 and *q* < 0.001 were defined as differentially expressed genes (DEGs). To further understand the functions of DEGs and the related biological processes they have a role in, gene ontology (GO) and KEGG analyses were conducted. The GO analysis classified DEGs into three categories: “molecular function”, “cellular component”, and “biological process” with a total of 45 GO terms (Figure 4A). The enriched GO terms were “binding” and “catalytic activity” within molecular function, “cell part”, “cell”, and “organelle” within cellular component and “cellular process”, “metabolic process”, and “response to stimulus” within biological process. The pathway enrichment analysis of the DEGs using KEGG identified significantly enriched “metabolic pathways” and “biosynthesis of secondary metabolites” (Figure 4B). As a result, the transcription analysis also showed that mycorrhizal fungus AR2 significantly affected the metabolic pathways in *A. roxburghii*.

### 2.5. Association Analysis of the DAMs and DEGs

**Figure 5:**
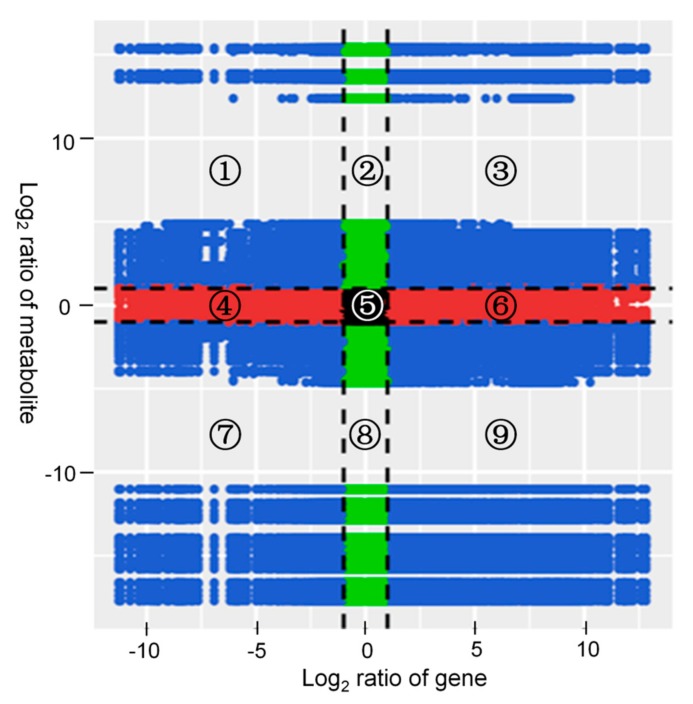
Quadrant diagrams representing the association of the DAMs and DEGs between the non-mycorrhizal and mycorrhizal *A. roxburghii* growth for six months. The x-axis represents that the log_2_ ratio of gene and the y-axis represents the log_2_ ratio of metabolite; black dotted lines represent the different threshold; each point represents a gene or metabolite; black dots represent the unchanged genes or metabolites; green dots represent differentially accumulated metabolites with unchanged genes; red dots represent differentially expressed genes with unchanged metabolites; blue dots represent both differentially expressed genes and differentially accumulated metabolites; it is divided into ①–⑨ quadrants from left to right and from top to bottom with black dotted lines; the ①, ② and ④ quadrants indicate that the expression abundance of metabolites is higher than that of genes; the ③ and ⑦ quadrants indicate that the expression patterns of genes are consistent with the metabolites; the ⑤ quadrant indicates that the genes and metabolites are not differentially expressed; the ⑥, ⑧ and ⑨ quadrants indicate that the expression abundance of metabolites is lower than that of genes.

Correlation analysis was carried out on the DAMs and DEGs. The variations in the metabolites and their corresponding genes with the Pearson correlation coefficient over 0.8 were selected to draw nine quadrant diagrams and the correlation coefficient cluster heat map. As shown in Figure 5, the higher number of DAMs and DEGs was in the seventh and ninth quadrants; and they were positively correlated in the seventh quadrant and negatively correlated in the ninth quadrant.

### 2.6. Dynamic Variations in Flavonoid DAMs

**Figure 6:**
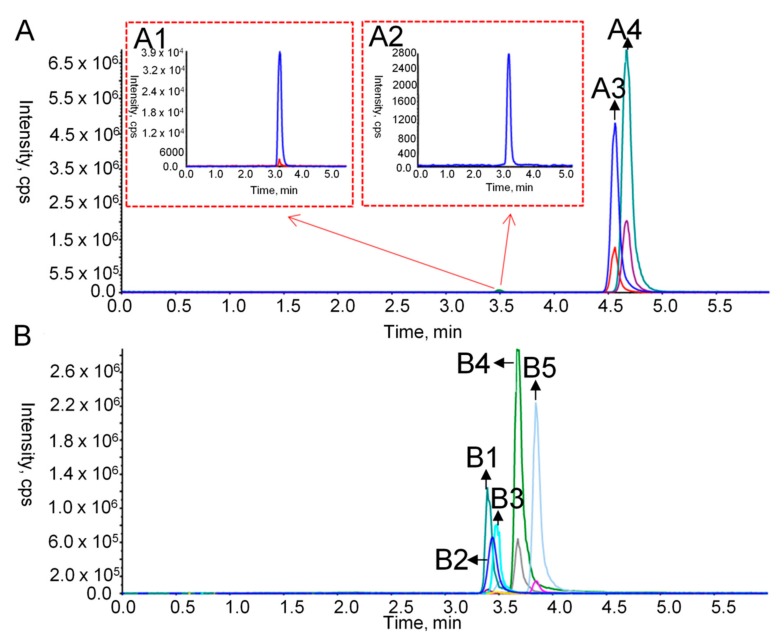
TIC diagrams of nine flavonoid standards in the positive and negative ion mode. (**A**) the positive ion mode; A1 represents the chromatograms of narcissin (retention time = 3.51 min); A2 is the ones of isorhamnetin-3-*O*-beta-d-glucoside (retention time = 3.53 min); A3 is the ones of nobiletin (retention time = 4.56 min); A4 is the ones of tangeretin (retention time = 4.67 min); (**B**) the negative ion mode; B1 represents the chromatograms of quercetin-7-*O*-glucoside (retention time = 3.43 min); B2 is the ones of rutin (retention time = 3.44 min); B3 is the ones of kaempferol-3-*O*-glucoside (retention time = 3.50 min); B4 is the ones of quercetin (retention time = 3.69 min); B5 is the ones of isorhamnetin (retention time = 3.84 min).

In order to measure the dynamic variations of nine flavonoid DAMs in M and NM at different growth stages, the extracts were detected using HPLC-MS/MS. The optimized parameters for each analyte are shown in Table 2. Figure 6 showed the TIC diagrams of nine standards in the positive and negative ion mode. The method validation including linearity, limit of detection (LOD), limit of quantification (LOQ), stability, precision, and repeatability is shown in Table 3 with an *R*^2^ higher than 0.9901, LOD ranging from 0.488 ng/mL to 15.63 ng/mL, LOQ ranging from 0.977 ng/mL to 31.25 ng/mL, the relative standard deviations (RSDs) of the stability ranging from 1.94% to 4.22%, the RSDs of the precision ranging from 1.43% to 4.49%, and the RSDs of the repeatability in the range of 1.74% to 4.93%. Additionally, the recovery rate was determined by adding 50%, 100% and 150% of the standard content of the sample. The average recovery rate of nine standards was from 86.03% to 99.54% with RSD between 1.09% and 4.55% (Table S2). These data indicated the reliability of the method.

**Figure 7:**
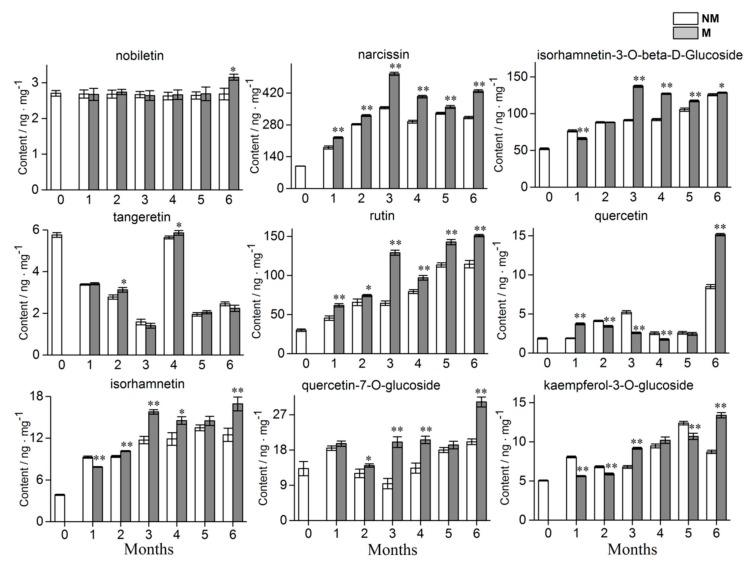
Dynamic variations of 9 flavonoids in the mycorrhizal and non-mycorrhizal *A. roxburghii* growth 0 month to 6 months. NM and M represents non-mycorrhizal *A. roxburghii* and mycorrhizal *A. roxburghii*, respectively. Each value is the mean of three replicates, and error bars indicate standard deviations. Statistical analysis of the data was performed by independent samples *t*-test using the SPSS 22.0 software (IBM, Chicago, IL, USA). * and ** above the columns are significantly different at *p* ≤ 0.05 and *p* ≤ 0.01, respectively.

For M and NM growth for six months, the tangeretin content was inhibited, while the content of the other 8 flavonoid DAMs was significantly upregulated which was consistent with the results of the metabolome and showed that the metabolome data were reliable. The levels of four metabolites (i.e., isorhamnetin-3-o-beta-d-glucoside, rutin, isorhamnetin, and kaempferol-3-*O*-glucoside) gradually increased across the growth time. For narcissin, quercetin, and quercetin-7-*O*-glucoside, the content tended to increase at first, then decrease and then increase again. While the tangeretin content reached the peak of accumulation at day 0 and the fourth month. Flavonoid accumulation in the plantlets showed significant difference between the fungal and no-fungal inoculations. The AR2 had a positive effect on narcissin, rutin, quercetin, and quercetin-7-*O*-glucoside content in *A. roxburghii* growth for one month, while it inhibited the accumulation of isorhamnetin-3-*O*-beta-d-glucoside, isorhamnetin, and kaempferol-3-*O*-glucoside and had no significant effect on nobiletin and tangeretin. Compared with the control group, significantly higher amount of narcissin, rutin and quercetin-7-*O*-glucoside accumulated in mycorrhizal plantlets from the first month to the sixth month. Up until the sixth month, AR2 could significantly promote the accumulation of nobiletin, but there was no significant difference at other times. The detailed results are shown in Figure 7. In conclusion, AR2 could significantly affect the accumulation of different flavonoids in *A. roxburghii*.

### 2.7. Dynamic Variations of Expression Levels of Flavonoid Biosynthetic Genes

**Figure 8:**
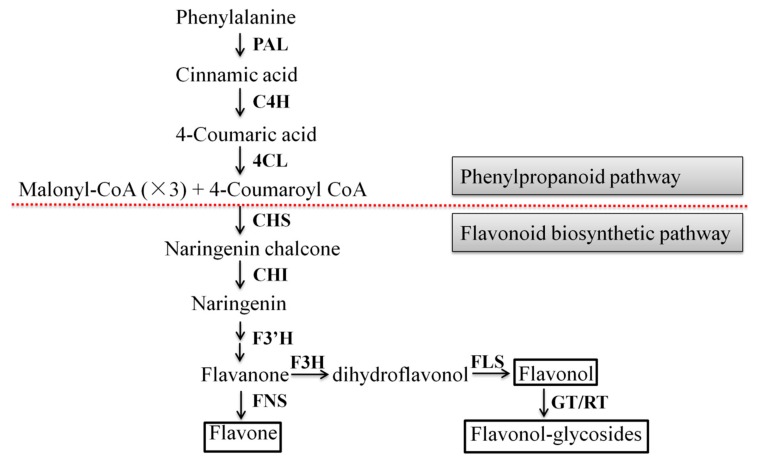
The flavonoid biosynthetic pathway in *A. roxburghii*. Bold words indicate the key enzymes in flavonoid biosynthesis. Compounds in the box show flavones, flavonols and flavonol-glycosides studied in this study.

**Figure 9:**
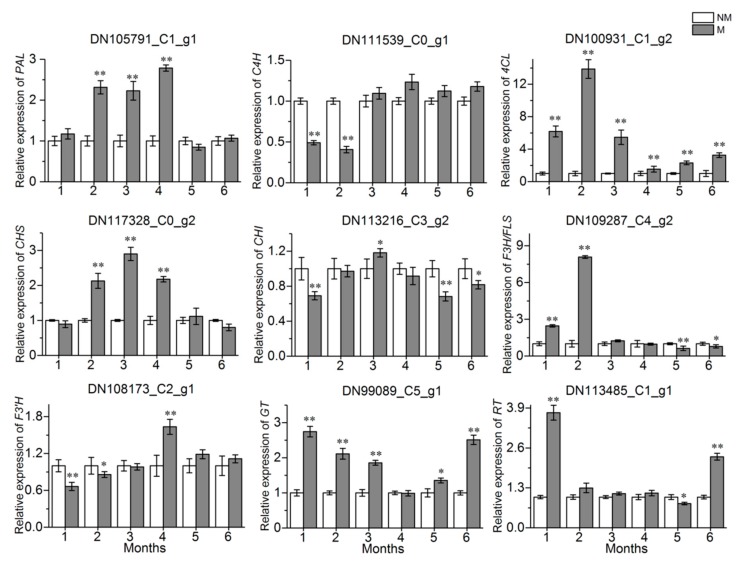
Expression levels of flavonoid biosynthetic genes in the M and NM growth 0 month to 6 months. M and NM represent the mycorrhizal *A. roxburghii* and non-mycorrhizal *A. roxburghii*, respectively. DNxx_Cx_g1 represents the ID of gene. The x-axis indicates the relative expression level of the genes. Each value is the mean of three replicates, and error bars indicate standard deviations. Statistical analysis of the data was performed by independent samples *t*-test using the SPSS 22.0 software (IBM, Chicago, IL, USA). * and ** above the columns are significantly different at *p* ≤ 0.05 and *p* ≤ 0.01, respectively.

The metabolic pathways of three common flavonoids in *A. roxburghii* are shown in Figure 8. qRT-PCR was used to assess the relative expression levels of nine key enzyme genes in the conserved flavonoid biosynthesis pathway in every treatment group. The results showed that the expression of the *4CL* gene in the treatment group was significantly upregulated during the whole growth process, compared with the control group (Figure 9). The expression of the *PAL* and *CHS* genes in the M was significantly upregulated at the same growth stage (2 months, 3 months, and 4 months), while it was almost the same at other growth stages. The AR2 showed an inhibitory effect on the expression of the *C4H* and *F3′H* genes in the 1 month old and 2 month old *A. roxburghii*, while it showed an inhibitory effect on the expression of the *CHI* gene in the 1 month old, 5 month old and 6 month old *A. roxburghii*. The *F3H/FLS* gene had an upregulated expression in the M grown for 1 month and 2 months and the *GT* gene also had an upregulated expression in the M grown for 1 month, 2 months, 3 months, 5 months, and 6 months. Finally, the expression of the *RT* gene was upregulated in the M grown for 1 month and 6 months. In conclusion, AR2 could induce the expression of flavonoid synthesis related genes in varying degrees and at different growth stages.

## 3. Discussion

Symbiotic association of mycorrhizal fungi with plants has been shown to affect flavonoid content. The application of co-cultivation of plants with orchid mycorrhizal (OM) fungi and arbuscular mycorrhizal (AM) fungi are progressing gradually. For AM fungi, they can penetrate and colonize the root of the host to form intracellular haustoria-like structures known as arbuscules, which are the principal sites of metabolic exchange between the two organisms [39,40]. Flavonoid content in *Medicago truncatula* was increased by AM inoculation [41]. Xie et al. [42] reported that AM colonization in the soybeans attributed to the increase of certain flavonoids in the root exudates. For OM fungi, they can form pleloton inside the root cells. And the association of OM with the orchid is the focus of our laboratory. Some studies indicated that flavonoid accumulated significantly in the mycorrhizal orchidaceae [16,43]. However, the dynamic changes of flavonoid content in mycorrhizal host at different developmental stages have rarely been studied. In our study, AR2 was belonging to a member of the orchid mycorrhizal fungi, and the co-cultivation between *A. roxburghii* and AR2 was performed. The dynamic changes of several flavonoids showed that the flavonoid had its special accumulation content at a defined growth time and that AR2 had different effects on different flavonoids at different growth stages. Also, AR2 induced the narcissin, rutin and quercetin-7-o-glucoside accumulations in mycorrhizal plantlets across the growth stage. For narcissin and isorhamnetin-3-o-beta-d-glucoside, their content in mycorrhizal *A. roxburghii* growth at three months reached the highest and was more than 420 ng/g and 120 ng/g, respectively. This was the first report regarding the changes of flavonoid content induced by AR2 in *A. roxburghii* at different growth stages.

To investigate the effects of mycorrhizal fungi on metabolites in its host, transcriptome and metabolome analyses were performed. Zhao et al. [29] reported that the secondary biosynthesis and hormone balance in the *Cymbidium hybridum* were induced by mycorrhizal fungus through transcriptome analysis. Schliemann et al. [44] reported that the biosynthesis of some constitutive isoflavonoids and plastidial metabolism could be activated by mycorrhizal fungus *Glomus intraradices* through metabolome analyses. In our study, metabolome analysis revealed that all 709 metabolites and 135 DAMs were putatively annotated among the NM and M. Among them, 148 flavonoid metabolites and 9 flavonoid DAMs were investigated. Furthermore, transcriptome analysis revealed that 4341 DEGs were identified between the two groups, of which 2915 DEGs were up-regulated and 1426 DEGs were down-regulated; KEGG pathways of the more DEGs were involved in the biosynthesis of secondary metabolites including flavonoid. These results implied that AR2 might change internal metabolism in *A. roxburghii*, especially for flavonoids, which would provide a basis for further study on the molecular mechanisms of AR2 promoting the flavonoid accumulation in *A. roxburghii*.

PAL, a key enzyme in the first step of the phenylpropanoid biosynthetic pathway, could be activated by fungal elicitors. Our study also revealed that the *PAL* gene had a significant upregulation, especially in the 2, 3, and 4 month mycorrhizal herbs, compared with the uninoculated ones. This result is in agreement with the results of Zhou et al. [45] and Xu et al. [46]. It is worth mentioning, the expression of the *4CL* gene in the plantlets inoculated AR2 during the whole growth process was significantly upregulated, with the highest expression being 13.3 fold. This is also consistent with Wang et al.’s report [20]. These data imply that AR2 might activate the downstream pathways of phenylpropanoids including flavonoids.

In addition, CHS, is the key enzyme in the flavonoids synthesis pathway [47]. Harrison and Dixon [48] reported that the expression level of the gene *CHS* in the roots of *Medicago truncatula* was enhanced by mycorrhizal fungus *Glomus versiforme*. Xie et al. [49] reported that mycorrhizal symbiosis induced the expression of the *CHS* gene of *Glycyrrhiza uralensis*, and the liquiritin accumulation and the expression of *CHS* gene showed a positive correlation. In our study, the expression level of the *CHS* gene was also upregulated in the mycorrhizal herbs growth for 2, 3, and 4 months; Meanwhile, the corresponding flavonoids (narcissin, rutin, isorhamnetin and quercetin-7-*O*-glucoside) accumulated in different degrees. Our data added new evidence to support mycorrhizal symbiosis induced the expression of the *CHS* gene and promoted the flavonoids accumulation. Additionally, our study showed that the *GT* gene expression was significantly upregulated in the 1–4 month mycorrhizal herbs, while the *RT* gene was induced in the 1–6 month ones. The corresponding flavonol-glycoside (narcissin, rutin, isorhamnetin-3-*O*-beta-d-glucoside, quercetin-7-*O*-glucoside and kaempferol-3-*O*-glucoside) showed basically the same induction trend in mycorrhizal *A. roxburghii*. These data again indicated that AR2 might activate the metabolic pathway of flavonoids.

In summary, this study provides much information about the changes that occur in the main active ingredient flavonoids and its related genes during different growth stages in M and NM. AR2 has different induction effects on flavonoid content and gene expression in *A. roxburghii* at different growth stages. These will provide a theoretical basis for reasonable harvest time of *A. roxburghii* and a new insight into improving the quality of the *A. roxburghii*.

## 4. Materials and Methods

### 4.1. Plant and Mycorrhizal Fungus Materials

Tissue culture plantlets of *A. roxburghii* were about 3 month-old and 2–3 cm in height from Yongan city, Fujian province, China. The mycorrhizal fungus AR2 (NCBI accession No: MN068847) was previously isolated in our laboratory and deposited at the microbiological center of the Institute of Medicinal Plant Development, Chinese Academy of Medical Sciences and Peking Union Medical College, Beijing, China. Before symbiotic cultures, the fungal strain was inoculated on the potato dextrose agar medium (PDA: potato 200 g·L^−1^, glucose 20 g·L^−1^, agar 12 g·L^−1^, pH 5.2) in darkness at a temperature of 25 ± 1 °C for 5 days. Two pieces (0.5 cm^3^) of the fungal inoculum were inoculated on solid matrix medium (sawdust: wheat bran: water = 3:1:1.5, *v/v/v*; 101.33 kPa and 121 °C for 180 min) in darkness at a temperature of 25 ± 1 °C for 15 days. After that, the fungal solid substrates were obtained for subsequent symbiotic culture.

### 4.2. Symbiotic Cultures of A. roxburghii Plantlets

In symbiotic cultures of plantlets, the substrate (humus soil: vermiculite: water = 3:1:1) was used. Each culture bottle (9 cm in diameter, 12.5 cm in height) containing 70 g substrate was sterilized at 101.33 kPa and a temperature of 121 °C for 180 min. Six plantlets, derived from asexual reproduction, were transferred into each culture bottle which was inoculated with 0.5 g of fungal solid substrates, while the culture bottles without fungal inoculum served as the control. The symbiotic cultures were placed in the growth room under a 12/12 h photoperiod at a temperature of 25 ± 1 °C and an illumination intensity of 1500 Lx. The samples (the whole plantlet) were collected once a month from the beginning of co-culture until the sixth month. Samples were immediately frozen in liquid nitrogen and stored at −80 °C for later analyses using LC-MS/MS, RNA-seq and qRT-PCR. Among them, the collected samples grown for six months were used for the metabolome and transcriptome analyses. All data were obtained based on three independent biological replicates.

### 4.3. Histological Study

Two weeks after inoculation, fresh root segments were fixed in 50 mM phosphate buffer (pH 6.8) containing 2.5% glutaraldehyde and 1.6% paraformaldehyde for 4 h at room temperature. After fixation, the samples were rinsed three times with phosphoric acid buffer (pH 6.8, 0.1 M) for about 15 min each time and then dehydrated with a graded ethanol series (15% ethanol for 30 min; 30% ethanol for 30 min; 50% ethanol for 30 min; 70% ethanol for 1 h; 85% ethanol for 1 h; 95% ethanol for 1 h; absolute ethanol for 1 h). After dehydration, the samples were embedded in LRwhite gradient mixture (25% LRwhite for 24 h; 50% LRwhite for 24 h; 75% LRwhite for 24 h; 100% LRwhite for 24 h) and polymerized for 48 h at 60 °C. Next, three-µm-thick sections were cut using glass knives with a rotary microtome (Autocut 2040; Reichert-Jung; Germany). The sections were collected on slides and stained with 0.05% (*w*/*v*) toluidine blue O in benzoate buffer for general histology examinations. The sections were examined, and images were captured digitally using a digital camera attached to the microscope (Axio Imager A1; Carl Zeiss, Oberkochen, Germany).

### 4.4. Sample Extraction and Metabolome Analysis

#### 4.4.1. Sample Extraction

The cryopreserved samples were freeze-dried and then ground for 1 min at 30 Hz using a MixerMill MM400 (Retsch Technology, Haan, Germany). Subsequently, 100 mg powder was weighed and extracted for 24 h at 4 °C in 1.0 mL of 70% methanol. During this period, the samples were vortexed (10 s, 40 Hz) once per 10 min for a total of three times. After extraction, the pellets were centrifuged at 10,000× *g* for 10 min at 4 °C and the extracts were then filtered through a 0.22 μm microporous membrane and stored in a sample vial. The QC was prepared by mixing all the samples. In order to examine the repeatability of the analysis process, a QC sample was injected after every five test samples during the instrumental analysis.

#### 4.4.2. Liquid Chromatographic Mass Spectrometry Analysis

The sample extracts were analyzed using a UPLC-ESI-MS/MS system, which mainly includes UPLC (Shim-pack UFLC SHIMADZU CBM30A, SHIMADZU, Kyoto, Japan) and MS/MS (Applied Biosystems 6500 QTRAP, AB SCIEX, Foster City, CA, USA). The UPLC separation was completed on a Waters Acquity UPLC HSS T3 C18 column (100 × 2.1 mm, 1.8 μm) (Waters Corp., Milford, MA, USA). The mobile phase A solvent was water containing 0.04% acetic acid, and the mobile phase B solvent was acetonitrile containing 0.04% acetic acid. The elution gradient was shown as follows: 0–11 min 95–5% A, 11–12 min 5–5% A, 12–12.1 min 5–95% A and 12–15 min 95–95% A. The flow was 0.4 mL/min and the injection volume was 2 μL. The column temperature was set to 40 °C.

The effluents were alternatively connected to an electrospray ionization-triple quadrupole-linear ion trap MS/MS (ESI-QTRAP-MS/MS). Linear ion trap (LIT) and triple quadrupole (QQQ) scans were carried out using QTRAP. The mass spectrometry conditions were as follows: the ESI temperature was set to 500 °C, the mass spectrometry voltage was 5500 V, the GS 1 and GS 2 were set to 55 psi and 60 psi, respectively, and the CUR was set to 25 psi. QQQ scans were obtained as MRM experiments with collision gas (nitrogen) set to 5 psi. The DP and CE for individual MRM transitions were optimized in the QQQ. The data were analyzed by the mass spectrometry software (Version 1.6.1 Applied Biosystems Company, Framingham, MA, USA).

#### 4.4.3. Metabolite Identification

Based on the public database of metabolite information and the MVDB V2.0 database of Wuhan Metware Biotechnology Co., Ltd. (Wuhan, China), qualitative analysis of the primary and secondary mass spectrometry data was performed by referencing the existing mass spectrometry databases such as MassBank, KNAPSAcK, HMDB and METLIN; thus, the structural analysis of metabolites was determined. Regarding the quantitative analysis of the metabolites, MRM was used, and PCA and OPLS-DA were then carried out to identify differential metabolites. A VIP ≥ 1 and a fold change ≥ 2 or ≤0.5 were set for metabolites with significant difference.

### 4.5. Illumina Sequencing

#### 4.5.1. RNA Extraction, cDNA Library Construction and Sequencing

The total RNA was extracted from NM and M using the RNeasy Plant Mini Kit (Qiagen, Hilden, Germany) following the manufacturer’s instructions, and treated with an RNase-free DNase I digestion kit (Aidlab, Beijing, China) in order to remove contaminated genomic DNA. RNA degradation was measured using 1% agarose gel, RNA concentration was measured using NanoDrop 2000 spectrophotometer (Thermo Scientific, Wilmington, DE, USA) and RNA integrity was assessed on an Agilent 2100 Bioanalyzer (Agilent, Palo Alto, CA, USA).

mRNA was enriched with oligo (dT)-attached magnetic beads and then the fragmentation buffer was exploited to randomly fragment the mRNA into short fragments. Using these cleaved RNA fragments as a template, the first cDNA strand was synthesized by random hexamers, then the second cDNA strand was synthesized by adding buffer, dNTPs, RNaseH and DNA polymerase I and purification of the double-stranded cDNA was done by NEBNext Ultra RNA Library Prep Kit for Illumina (NEB, Ipswich, MA, USA). The purified double-stranded cDNA was repaired by end-to-end, added poly A tail and connected to the sequencing connector. cDNA of about 200 bp in length was selected by AMPure XP beads, and PCR amplification was performed to enrich the purified cDNA template. Finally, the libraries were sequenced using an Illumina Hiseq 2000. Sequence data were deposited in the NCBI SRA database (accession number: PRJNA579778).

#### 4.5.2. De Novo Transcriptome Assembly and Annotation

In order to achieve high-quality and clean data, the raw data was filtered by removing the reads with adapter sequence and low-quality reads using the fastp software [50], then the clean reads were assembled into transcriptome by the Trinity v.2.0.6 software (Broad Institute, Cambridge, MA, USA). Unigene function was annotated based on these databases: NCBI non-redundant protein sequences (Nr, https://ftp.ncbi.nlm.nih.gov/blast/db/FASTA/), KEGG (https://www.genome.jp/kegg), Clusters of Orthologous Groups of proteins (COG, https://www.ncbi.nlm.nih.gov/COG/), GO (https://www.geneontology.org), the Swiss-Prot (http://www.ebi.ac.uk/uniprot/) and the translation of EMBL (TrEMBL).

### 4.6. Determination of the Flavonoid Contents During Different Growth Stages

#### 4.6.1. Preparation of Standard Solutions

In order to validate the changes of flavonoids during different growth stages after symbiotic culture, several monomer flavonoids from DAMs were tested. The standards of nobiletin (478-01-3), narcissin (604-80-8), isorhamnetin-3-*O*-beta-d-Glucoside (5041-82-7), tangeretin (481-53-8), rutin (153-18-4), quercetin (117-39-5), isorhamnetin (480-19-3), quercetin-7-*O*-glucoside (491-50-9), and kaempferol-3-*O*-glucoside (480-10-4) were purchased from the National Institutes for Food and Drug Control (Beijing, China) and the purity of all of them was over 98%. Each standard sample was dissolved in methanol to obtain 1 μg/mL of standard working solutions and used within 1 month at −20 °C.

#### 4.6.2. Preparation of Sample Solutions

The preparation of the sample (approximately 20 mg) followed the same process as the above mentioned extract. In order to enrich the flavonoids, the extract was processed through C18-SPE solid phase extraction column (Waters Technology Co., Ltd., USA) and the column was rinsed with 10%, 20%, 30%, 40%, 50%, 60%, 70%, 80%, and 90% absolute alcohol. The rinse solutions of 70%, 80%, 90%, and absolute alcohol, that were verified to contain flavonoids, were collected and freeze dried using a vacuum freeze-drying machine LGJ-18 (Beijing Songyuanhuaxing Technology Develop Co., Ltd, Beijing, China). The extract was re-dissolved with 2 mL methanol, then the sample solution was passed through a 0.22 μm syringe nylon filter and stored at −20 °C prior to analysis.

#### 4.6.3. Apparatus and Analytical Conditions

The HPLC separation was performed by an Agilent 1260 Infinity II series HPLC System that was equipped with Waters Atlantis C18 column (150 × 3.9 mm, 5 μm). The mobile phase consisted of solvent A (water containing 0.1% formic acid) and solvent B (methanol). The gradient procedure was as follows: 0–3 min 60–5% A, 3–5 min 5–5% A, 5–5.02 min 5–60% A, and 5.02–6 min 60–60% A. The flow rate was 0.5 mL/min and the injection volume was 5 μL.

An Applied Biosystems Sciex QTRAP® 4500 MS/MS spectrometer equipped with a version of 1.6 Analyst software (AB SCIEX, Massachusetts, USA) was used for the analysis. The instrument was equipped with an ESI source, and the targeted analytes were performed in positive and negative ion modes for all the targeted analytes. Compressed air was used as GS1 and GS2, and high-purity (99.99%) nitrogen was used as CUR and CAD. The operation conditions were as follows: the EP was 10.0/−10.0 V, the TEM at 500 °C, the IS 5500/–4500 V, GS1 set to 55 psi, GS2 set to 55 psi, CUR set to 35 psi, and the CXP 13.0/−15.0 V for ESI^+^/ESI^−^ mode, respectively. The dwell time for each MRM transition was 10 ms.

### 4.7. Expression of the Flavonoid Biosynthesis Related Genes During Different Growth Stages

Nine unigenes that were related to flavonoid biosynthesis following Zhang et al. [51] and Park et al. [52] were selected for validation using qRT-PCR. The RNA was reverse-transcribed to cDNA using PrimeScriptTM RT reagent Kit (TaKaRa, Dalian, China). The qRT-PCR was performed using the SYBR®Premix ExTaq TM (TaKaRa, Dalian, China) on the LightCycler® 480 II Real-Time PCR System (Roche, Carlsbad, CA, USA). Three biological replicates and three technical replicates were performed, and all the primer names and corresponding sequences are listed in Table S3. The qRT-PCR was performed in a 20 μL reaction volume containing SYBR Premix Ex Taq II (10 μL), forward prime (10 μM, 0.8 μL), reverse primer (10 μM, 0.8 μL), cDNA template (5 ng/μL, 2 μL), and ddH_2_O (6.4 μL). The PCR conditions were as follows: denaturation at 95 °C for 30 s, followed by 40 cycles of amplification (95 °C for 5 s, 60 °C for 30 s). The melting curves were measured at 95 °C for 5 s and 60 °C for 1 min. The elongation factor 1 alpha (*EF-1α*) gene of *A. roxburghii* was used as the internal control reference gene. Finally, gene expression was calculated using the 2^−ΔΔCt^ method [53].

### 4.8. Statistical Analysis

All the transcriptome and metabolome samples were designed for three biological replicates. Statistical analyses were conducted using the SPSS 22.0 software (IBM, Chicago, IL, USA). An independent samples *t*-test was used for statistical evaluations between the control and treatment groups.

## Figures and Tables

**Table 1:** Summary of the analysis of transcriptome sequences from the NM and M growth for six months.

Sample	Raw Reads	Clean Reads	Clean Base (G)	Error Rate (%)	Q20 (%)	Q30 (%)	GC Content (%)
NM1	61,226,728	61,071,914	9.09	0.017	97.79	93.61	48.19
NM2	60,542,772	60,425,910	8.99	0.016	98.11	94.42	47.93
NM3	67,559,786	67,410,292	10.05	0.016	98.07	94.28	48.43
M1	55,632,192	55,492,010	8.28	0.016	98.02	94.20	49.06
M2	65,007,376	64,859,884	9.68	0.016	98.19	94.58	48.10
M3	67,125,158	66,965,132	9.99	0.016	98.09	94.34	48.23

NM: represents non-mycorrhizal *A. roxburghii*; M: represents mycorrhizal *A. roxburghii.*

**Table 2:** The optimized parameters for nine flavonoid standards by HPLC-MS/MS.

No.	Name	Precursor Ion (*m*/*z*)	Product Ions (*m*/*z*)		DP (V)	CE (V)	EP (V)	CXP (V)	IS (V)	Ionization Mode	Retention Time (min)
			PI ^q^	PI ^i^							
1	nobiletin	402.9	373.1	388.1	20	33	10	13	5500	ESI^+^	4.56
2	narcissin	624.9	316.7	479.6	50	25	10	13	5500	ESI^+^	3.51
3	isorhamnetin-3-*O*-beta-d-glucoside	479.3	317	253	150	34	10	13	5500	ESI^+^	3.53
4	tangeretin	372.8	343.1	358.1	10	32	10	13	5500	ESI^+^	4.67
5	rutin	609	299.9	279.5	−50	−43	−10	−15	−4500	ESI^−^	3.44
6	quercetin	301	150.8	178.9	−100	−35	−10	−15	−4500	ESI^−^	3.69
7	isorhamnetin	314.8	300	150.8	−150	−30	−10	−15	−4500	ESI^−^	3.84
8	quercetin-7-*O*-glucoside	462.9	300.9	342.9	−50	−28	−10	−15	−4500	ESI^−^	3.43
9	kaempferol-3-*O*-glucoside	592.9	284.9	255	−50	−40	−10	−15	−4500	ESI^−^	3.50

DP de-clustering potential; EP entrance potential; CXP collision cell exit potential; CE collision energy; PI product ions; ^q^ for quantification; ^i^ for identification; CUR curtain gas; IS ion spray voltage; TEM temperature; GS1 ion source gas 1; GS2 ion source gas2; CUR = 35 psi; TEM = 500 °C; both GS1 and GS2 = 55 psi.

**Table 3:** Method validation results including linearity, LOD, LOQ, stability, precision and repeatability.

No.	Name	Linearity			LOD (ng/mL)	LOQ (ng/mL)	Stability (RSD, %)	Precision (RSD, %)	Repeatability (RSD, %)
		Regression Equations	R^2^	Ranges (ng/mL)					
1	nobiletin	y = 62,293,800x − 1,576,600	0.9968	7.81–1000	0.488	0.977	3.05	1.43	4.44
2	narcissin	y = 3,029,650x − 314,384	0.9942	31.25–4000	7.81	15.63	3.26	3.22	2.26
3	isorhamnetin-3-*O*- beta-d-glucoside	y = 279,387x − 11,500	0.9973	62.5–4000	15.63	31.25	4.22	4.37	4.93
4	tangeretin	y = 4,034,570x − 18,086	0.9902	3.91–250	0.488	0.977	1.94	1.76	4.87
5	rutin	y = 3,089,740x − 61,316	0.9978	15.63–1000	7.81	31.25	2.99	3.71	1.74
6	quercetin	y = 17,556,400x − 169,184	0.9921	15.63–500	7.81	15.63	3.82	4.03	4.9
7	isorhamnetin	y = 116,434,300x − 149,754	0.9903	7.81–250	3.91	7.81	2.53	2.94	2.82
8	quercetin-7-*O*- glucoside	y = 5,857,970x − 144,514	0.9989	31.25–1000	7.81	15.63	3.26	3.31	2.39
9	kaempferol-3-*O*- glucoside	y = 4,499,260x − 117,172	0.9901	31.25–1000	7.81	15.63	2.06	4.49	4.9

## Supplementary Materials

Supplementary materials can be found at https://www.mdpi.com/1422-0067/21/2/564/s1. Figure S1: Overlapping analysis of the TIC in QC samples. The x-axis represents the retention time (min) of metabolite detection, and the y-axis represents the intensity of ion current (cps: count per second). (A) for ESI^+^ mode; (B) for ESI^−^ mode; Table S1: Total identified metabolites in the NM and M growth for six months; Table S2: Recovery test for each analyte; Table S3: Primer designed for qRT-PCR.

## Abbreviations

CE	collision energy
CUR	curtain gas
CXP	collision cell exit potential
DAMs	differentially accumulated metabolites
DEGs	differentially expressed genes
DP	de-clustering potential
EP	entrance potential
ESI-QTRAP-MS/MS	electrospray ionization-triple quadrupole-linear ion trap MS/MS
GC-MS	gas chromatography-mass spectrometer
GO	gene ontology
GS1	ion source gas 1
GS2	ion source gas 2
HPLC-MS/MS	high-performance liquid chromatography coupled with tandem mass spectrometry
IS	ion spray voltage
KEGG	Kyoto encyclopedia of genes and genomes
LC-MS	liquid chromatograph-mass spectrometer
LIT	linear ion trap
LOD	limit of detection
LOQ	limit of quantification
NMR	nuclear magnetic resonance
OPLS-DA	orthogonal partial least squares discriminant
PCA	principal component analysis
PC1	principal component 1
PC2	principal component 2
PI	product ions
QC	quality control
QQQ	triple quadrupole
qRT-PCR	quantitative real-time polymerase chain reaction
RNA-seq	RNA-sequencing
RSDs	relative standard deviations
TEM	temperature
TIC	total ion chromatography
UPLC-ESI-MS/MS	ultra-performance liquid chromatography-electrospray ionization-tandem mass spectrometry
VIP	variable importance in project
cps	count per second
MRM	multiple reaction monitoring
psi	pounds per square inch
NM	non-mycorrhizal *A. roxburghii*
M	mycorrhizal *A. roxburghii*
PAL	phenylalanine ammonia-lyase
C4H	cinnamate 4-hydroxylase
4CL	4-coumarate CoA ligase
CHS	chalcone synthase
CHI	chalcone isomerase
F3′H	flavonoid 3′-hydroxylase
FNS	flavone synthase
F3H	flavanone 3-hydroxylase
FLS	flavonol synthase
GT	flavonoid 3-*O*-glucosyltransferase
RT	rhamnosyltransferase
