# Supplementary material for: Combined Metabolome and Transcriptome Analyses Reveal the Effects of Mycorrhizal Fungus Ceratobasidium sp. AR2 on the Flavonoid Accumulation in Anoectochilus roxburghii during Different Growth Stages
Source: Int J Mol Sci. 2020 Jan 15;21(2):564. doi: 10.3390/ijms21020564 (PMC7013922; doi:10.3390/ijms21020564)
Supplement: Supplementary file 1 [file ijms-21-00564-s001.zip › Supplementary Figure S1.pdf]

Supplementary Figure

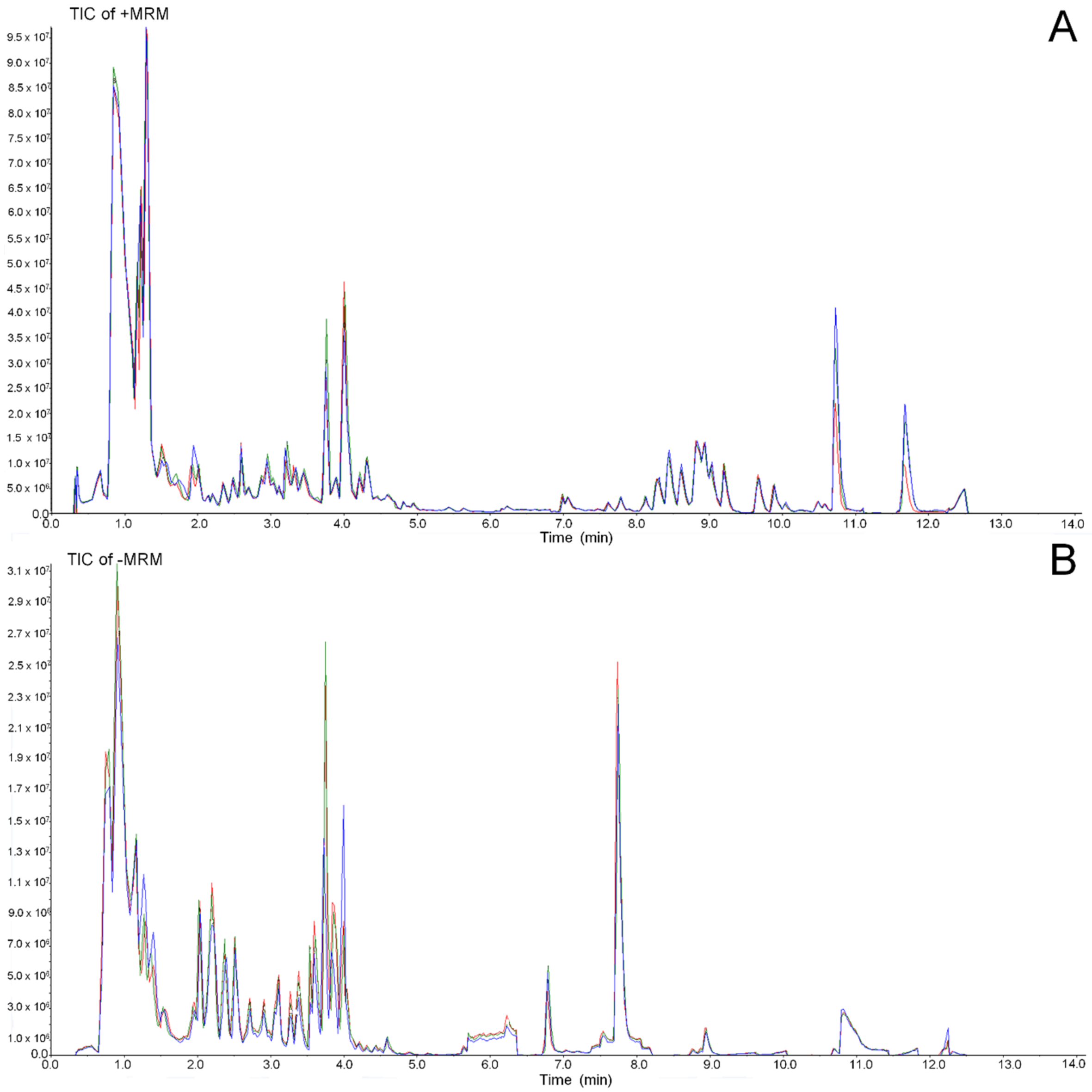

Figure S1. overlapping analysis of the TIC in QC samples. The x-axis represents the retention time (min) of metabolite detection, and the y-axis represents the intensity of ion current (cps: count per second). (**A**) for ESI^{+} mode; (**B**) for ESI^{-} mode.
